# Supplementary material for: Chinese residents' environmental concern and expectation of sending children to study abroad
Source: PLoS One. 2020 Sep 22;15(9):e0238180. doi: 10.1371/journal.pone.0238180 (PMC7508386; doi:10.1371/journal.pone.0238180)
Supplement: Attachment — Submitted filename: Response to reviewer.docx [file pone.0238180.s002.docx]

**Reviewer:**

Paper discusses an interesting connection possible between desires for children to study abroad and concerns about environment in China. While these two variables do not have immediate linkage, it is interesting to speculate about this connection and the study seems to have been done carefully using existing data from a survey. The topic would be of interest to many readers. One addition that would be valuable would be more information about the questions on "environmental concerns" as this is a very broad topic.

Reply: Dear Reviewer, thank you very much for the valuable advice. The following are my revision based on your advice.

The definition of environmental concern in the study has been presented and specified, as shown:

“**Environmental concern refers to awareness of high correlation between natural environment condition and life quality, such as perceived health risk of hazardous environment (e.g., air pollution, water pollution, garbage pollution). Environmental concern also implies people’s behavioural intentions for improving natural environment quality. For example, people with high environmental concern would like to pay for better natural environment in the context of feasible financial burden.**” (Line 5, P5)

**Editor**:

1."The study is well executed because the hypotheses were clearly stated, the data collection and analyses were appropriate, and the discussion of findings did not go beyond the study results. That is, the study followed appropriate protocols for such a study.

In regard to the issue of "environmental concerns" it would be useful to have the authors spell out in more detail what types of environmental concerns they are looking at. This is a very broad statement and the reader would be helped if the types of environmental concerns of interest to the authors were described in a few more sentences."

Reply: Dear Editor, thank you very much for the generous help. The following are my revision based on your advice.

As shown in the Line5, P5, “**Environmental concern refers to awareness of high correlation between natural environment condition and life quality, such as perceived health risk of hazardous environment (e.g., air pollution, water pollution, garbage pollution). Environmental concern also implies people’s behavioural intentions for improving natural environment quality. For example, people with high environmental concern would like to pay for better natural environment in the context of feasible financial burden**.” These sentences specify the implication of environmental concern.

2.In addition, I strongly recommend that you revise the english for your manuscript with the help of a native English proofreader.

Reply: Dear Editor, thanks for the kind reminding. I have invited AJE to proofread the manuscript as shown in the revised manuscript.
